# Copy number variation and regions of homozygosity analysis in patients with MÜLLERIAN aplasia

**DOI:** 10.1186/s13039-018-0359-3

**Published:** 2018-02-03

**Authors:** Durkadin Demir Eksi, Yiping Shen, Munire Erman, Lynn P. Chorich, Megan E. Sullivan, Meric Bilekdemir, Elanur Yılmaz, Guven Luleci, Hyung-Goo Kim, Ozgul M. Alper, Lawrence C. Layman

**Affiliations:** 1 Department of Medical Biology, Alanya Alaaddin Keykubat University, Faculty of Medicine, Antalya, Turkey; 2 Guangxi Maternal and Child Health Hospital, Nanning, China; 3 Department of Pathology, Harvard Medical School, Boston, MA 02115 USA; 4 Division of Genetics and Genomics, Boston Children’s Hospital, Boston, MA 02115 USA; 5 Shanghai Children’s Medical Center, Shanghai Jiaotong University School of Medicine, Shanghai, 200127 China; 6 Department of Obstetrics and Gynecology, Akdeniz University, Faculty of Medicine, Antalya, Turkey; 7 Section of Reproductive Endocrinology, Infertility, & Genetics, Department of Obstetrics & Gynecology Medical College of Georgia at Augusta University, Augusta, GA USA; 8 Department of Neuroscience & Regenerative Medicine, Medical College of Georgia at Augusta University, 1120 15th Street, CA2041, Augusta, GA 30912 USA; 9 Department of Medical Biology and Genetics, Akdeniz University, Faculty of Medicine, 07058 Antalya, Turkey

**Keywords:** Müllerian aplasia, Mayer-Rokitansky-Küster-Hauser syndrome, MRKH, Congenital absence of the uterus and vagina, Copy number variant, CNV, Candidate gene, Regions of homozygosity, ROH

## Abstract

**Background:**

Little is known about the genetic contribution to Müllerian aplasia, better known to patients as Mayer-Rokitansky-Küster-Hauser (MRKH) syndrome. Mutations in two genes (*WNT4* and *HNF1B*) account for a small number of patients, but heterozygous copy number variants (CNVs) have been described. However, the significance of these CNVs in the pathogenesis of MRKH is unknown, but suggests possible autosomal dominant inheritance. We are not aware of CNV studies in consanguineous patients, which could pinpoint genes important in autosomal recessive MRKH. We therefore utilized SNP/CGH microarrays to identify CNVs and define regions of homozygosity (ROH) in Anatolian Turkish MRKH patients.

**Result(s):**

Five different CNVs were detected in 4/19 patients (21%), one of which is a previously reported 16p11.2 deletion containing 32 genes, while four involved smaller regions each containing only one gene. Fourteen of 19 (74%) of patients had parents that were third degree relatives or closer. There were 42 regions of homozygosity shared by at least two MRKH patients which was spread throughout most chromosomes. Of interest, eight candidate genes suggested by human or animal studies (*RBM8A, CMTM7, CCR4, TRIM71, CNOT10, TP63, EMX2,* and *CFTR*) reside within these ROH.

**Conclusion(s):**

CNVs were found in about 20% of Turkish MRKH patients, and as in other studies, proof of causation is lacking. The 16p11.2 deletion seen in mixed populations is also identified in Turkish MRKH patients. Turkish MRKH patients have a higher likelihood of being consanguineous than the general Anatolian Turkish population. Although identified single gene mutations and heterozygous CNVs suggest autosomal dominant inheritance for MRKH in much of the western world, regions of homozygosity, which could contain shared mutant alleles, make it more likely that autosomal recessively inherited causes will be manifested in Turkish women with MRKH.

## Introduction

Approximately 7–10% of women have uterovaginal anomalies [1], but perhaps the most severe is Müllerian aplasia, which is also known as Mayer-Rokitansky-Küster-Hauser (MRKH) syndrome—the name patients prefer [2]. These patients have congenital absence of the uterus and vagina (type I; MIM# 277000), or they may also have associated anomalies such as renal agenesis, skeletal abnormalities, cardiac anomalies, or deafness (type II; MIM# 601076) [3]. Additionally, emotional issues as well as concerns regarding family planning are prevalent for these patients [4]. Although MRKH affects ~ 1/4500–1/5000 females, it accounts for about 10% of the causes of primary amenorrhea in females [5].

There is evidence for genetic transmission, as there are some families with more than one affected MRKH individual [6, 7]. In our recent characterization of both North American and Turkish families (*n* = 147 probands), no family had more than one affected individual, but some had another person with one or more of the associated anomalies [2]. Vertical transmission is challenging to confirm unless the MRKH woman conceive with IVF and use a gestational carrier. Consequently, the genetic etiology of MRKH is largely unknown. To date, only two genes—*WNT4* [8–11] and *HNF1B* [12]—have confirmed, causative mutations in a handful of MRKH patients. A total of four translocations have been identified in MRKH [13–15], but in only one were the breakpoints mapped [15]. Although no gene was directly disrupted, this valuable patient with a translocation involving chromosomes 3p22.3 and 16p13.3 can help pinpoint potential candidate genes that could be affected by a position effect [15].

A number of investigators have utilized chromosomal microarrays (CMAs) in MRKH either by comparative genomic hybridization (CGH) and/or single nucleotide polymorphism (SNP) techniques [16–21]. Reported copy number variants (CNVs) identified are abundant, but several have been found repetitively including deletions of 17q12, 16p11, and 22q11 [19]. Deletions and duplications of 1q21.1 have also been described by multiple investigators [16, 20, 22, 23]. These chromosomal regions contain numerous genes, and although they contain promising candidate genes, their role in causation is currently unknown. To date, all of the CNV studies in MRKH have been in mixed, nonconsanguineous, non-autosomal recessive populations. In the present study, we sought to use CMAs to identify CNVs and regions of homozygosity (ROH) in a suspected consanguineous Turkish population to provide additional clues to important candidate genes which might cause autosomal recessive MRKH.

## Methods

### Patients

Nineteen Anatolian Turkish patients with a normal 46,XX karyotype were diagnosed with MRKH in the Department of Obstetrics and Gynecology at Akdeniz University Hospital, Turkey and the study took place there and at the Medical College of Georgia at Augusta University, USA. The study was approved by the Institutional Review Boards at both locations, and each person signed a consent form. All patients had normal breast development and an absent vagina by exam supported by imaging studies. Of these 19, three had renal agenesis and two had hypoplastic ovaries (Table 1). Consanguinity was ascertained by family history when the patient was enrolled in the study. Genomic DNA was extracted from peripheral blood samples of patients and available family members by a non-enzymatic salt-precipitation method as described previously [24].

**Table 1:** The associated clinical findings in the MRKH cohort

Patient	Finding
3	Hypoplastic ovary
10	Unilateral Renal agenesis
14	Hypoplastic ovary
16	Unilateral Renal agenesis
17	Unilateral Renal agenesis

### Copy number variation (CNV) analysis

Copy number variant analysis was performed on all 19 patients and available family members (if a CNV was identified) with the use of an Affymetrix Cytoscan HD array (Affymetrix, Inc., Santa Clara, CA), which contains 750,000 single-nucleotide polymorphism probes and 1.9 million oligonucleotide probes. The lower limit of detection for CNVs was 50 kilobases (kb). One hundred nanograms of genomic DNA was labeled and used along with the Cytoscan reagent kit according to the manufacturer’s instructions. The array data were analyzed with Chromosome Analysis Suite software as described previously [25]. Human genome hg19 assembly was used to map genomic coordinates. The identified CNVs were compared with Database of Genomic Variants (DGV, http://projects.tcag.ca/cgi-bin/variation/gbrowse/hg19/) to determine if they were unique or previously identified. The CNVs were also investigated for potential pathogenicity using Decipher (https://decipher.sanger.ac.uk/).

### Analysis of parental consanguinity and regions of homozygosity

Patient history was used to ascertain degree of consanguinity in the parents of the MRKH subject. Regions of homozygosity (ROH) analysis was performed on all 19 Turkish patients tested using the Affymetrix Cytoscan HD platform. The degree of parental consanguinity was assessed according to the percentage of homozygosity (F_ROH_), which is also known as a coefficient of consanguinity. F_ROH_ was calculated by summing autosomal homozygous DNA basepairs (> 5 Mb includes at least 100 consecutive probes) and dividing by total basepair of autosomal genome DNA [25]. The percentage of autosome/genome homozygosity (CHP Summary) determined by F_ROH_ was analyzed using Chromosome Analysis Suite (ChAS) 1.2 software (Affymetrix Data Analysis Software). The thresholds of the percentage of ROH to predict the degree of consanguinity were taken from Sund et al. [25]. Overlapping homozygous genomic regions in at least two patients were determined by comparing the length of shared sequence.

## Results

Five different likely pathogenic CNVs were identified in four of 19 (21%) Turkish patients by CMA (Table 2), all of whom had isolated (type I) MRKH. One was the previously described 16p11.2 in MRKH, which was a 746 kb deletion, for which a similar sized CNV was seen in DGV six times, but not in Decipher. Note that when any sized CNV that overlaps the 16p11.2 region is considered, this was seen 125 times in DGV and 10 times in Decipher. This patient also had an Xq25 deletion of 768 kb present once in DGV, but not Decipher (any sized CNV 17 times in DGV; none in Decipher). Within the Xq25 deletion, there was only one gene. One patient had 16p13.3 deletion, which was present multiple times in both DGV and Decipher. The other two MRKH patients had duplications of 13q14.11 (once in DGV; not in Decipher) and 1p31.1 (not in DGV or Decipher) (Table 2). Except for the 16p11.2 deletion, which contained 39 genes, the other CNVs each only had 1–3 genes (Table 2). Family members for these four MRKH patients were not able to be studied, so it is not known if they are de novo.

**Table 2:** Shown are five different copy number variants (CNV) that were identified in four Turkish patients with type I MRKH

Patient	CNV Location	Size/Type	Coordinates	# times in DGV	# times in Decipher	Genes in CNV
6	16p11.2	746 kb Del	29,432,212–30,177,916	6 (125)	0 (10)	39
	Xq25	768 kb Del	126,937,856–127,706,114	8 (17)	0 (0)	1 (*ACTRT1*)
7	16p13.3	243 kb Del	6,774,500–7,017,793	Multiple (131)	Multiple [25]	1(*RBFOX1*)^*^
8	13q14.11	116 kb Dup	41,178,626–41,294,741	1 (12)	0 (0)	1 (*FOXO1*)
9	1p31.1	263 kb Dup	76,357,590–76,620,268	0 (19)	0 (0)	3 (*ST6GALNAC3*, *MSH4, ASB17*)

*DGV* Database of Genomic Variants, *Del* deletion, *Dup* duplication. The number of times a very similar sized CNV is listed for both DGV and Decipher. In parentheses, shown is the number of times a CNV of any size overlapped any portion of our CNV region

*RBFOX1 is a gene known in relation to autism. Only patient number 6 had parents who were not consanguineous (4th degree relatives). Patient numbers 7 and 8 had parents that were 3rd degree relatives, while patient 9 had parents that were 2nd degree relatives

By history, 11 of the 19 Turkish patients did not know if consanguinity was present, while eight stated that their parents were first cousins. First cousins should share 1/16 (6.25%) of sequence. When ROH were analyzed, the degree of consanguinity was greater than the patient previously reported (Table 3). Instead of parents being third degree relatives, six were found to be second degree relatives with sharing of 8.8–18.3% loci, one was first or second degree (20% shared loci), and one was first degree (23.5% shared loci). For the 11 for whom no history was known, parents were second degree in one and third degree in three, while the others were third or fourth degree relatives. In total, 14 of 19 (~ 74%) MRKH patients had parents that were third degree relatives or closer.

**Table 3:** Re-defined degree of consanguinity

	Before Analysis	After Analysis	
Patient	Parental Consanguinity (based on patient’s interview)	% Autosomal ROH	Parental Consanguinity Degree
1	No Info	3.7%	Fourth degree
2	No Info	2.9%	Fourth degree
3	First Cousins	10.3%	Second degree
4	First Cousins	10.7%	Second degree
5	First Cousins	11.4%	Second degree
6	No Info	4.0%	Fourth degree
7	No Info	6.86%	Third degree
8	No Info	5.8%	Third degree
9	No Info	9.9%	Second degree
10	No Info	4.4%	Third or fourth degree
11	No Info	3.7%	Fourth degree
12	No Info	13.1%	Second degree
13	First Cousins	18.3%	Second degree
14	First Cousins	8.8%	Second degree
15	First Cousins	14.7%	Second degree
16	First Cousins	20%	First or second degree
17	No Info	6.4%	Third Degree
18	First Cousins	23.5%	First degree
19	No Info	20.9%	Second degree
Consanguinity Degree	Theoretic Percentage	Percentage of Homozygosity (Confidence Interval)	
First or closer	> 25%	> 28.7%	
First	25%	21.3–28.7%	
First or second		15.3–21.3%	
Second	12.5%	9.7–15.3%	
Second or third		8.3–9.7%	
Third	6.25%	4.6–8.3%	
Third or fourth		4.2–4.6%	
Fourth	3.125%	2.6–4.2%	
Fourth or fifth		1.6–2.6%	
Fifth	1.5625%	0.5–1.6%	

**Fig. 1:**
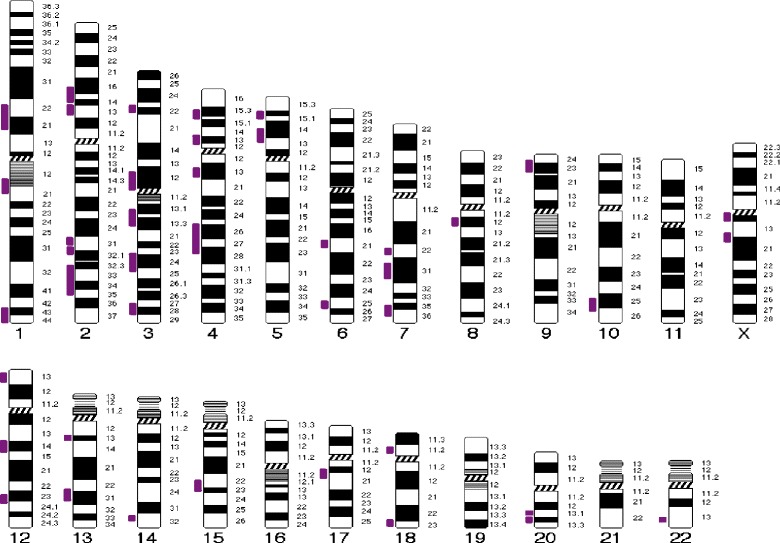
The 42 regions of homozygosity shared by at least two different Turkish MRKH patients are indicated to the left of each chromosome as a vertical bar

In addition, there were 42 regions across the genome in which at least two MRKH patients had overlapping homozygous genomic regions (Table 4 and Fig. 1). The most frequently shared chromosomes were chromosomes 2, 3, and 4. All chromosomes were represented except 11, 16, 19, and 21. The shared regions contained as few as 10 genes or as many as 354 genes. None of the shared regions included the more common 17q12 or 16p11.2 CNVs, but two shared the 22q11.21 CNV region (Table 4).

**Table 4:** Overlapping regions of homozygosity

Chromosome	Cytoband Start	Min (Hg19)	Max (Hg19)	Gene Count	Number of patients (n)	Candidate gene
1	p22.3	87,889,117	101,551,513	150	2	
1	q21.1	144,033,938	150,574,441	56	2	*RMB8A*
1	q43	242,177,676	249,198,692	354	2	
2	p16.3	49,466,260	65,782,717	246	3	
2	p14	67,193,897	74,970,256	23	3	
2	q24.3	171,534,387	175,330,938	45	2	
2	q31.1	192,319,867	217,837,588	237	2	
2	q31.1	177,426,525	185,333,874	342	2	*CMTM7, CCR4, TRIM71, CNOT10*
3	p12.3	76,456,413	90,485,635	67	2	
3	p24.3	31,161,056	36,796,647	89	2	
3	q11.1	102,994,376	115,492,735	321	3	
3	q23	139,702,339	150,629,667	234	2	
3	q26.31	187,040,042	190,991,439	65	2	*TP63*
4	p14	40,533,584	45,755,965	76	2	
4	p15.33	11,546,274	16,693,715	34	2	
4	q11	65,736,529	71,893,827	87	3	
4	q22.1	111,799,253	139,609,452	231	3	
5	p15.1	9,998,327	17,326,672	341	2	
5	p15.2	18,320,731	31,181,789	23	2	
6	q16.1	106,018,502	110,701,451	45	2	
6	q25.2	153,345,184	158,377,316	56	2	
7	q21.3	103,575,957	105,632,704	78	2	
7	q31.1	111,645,191	124,187,217	65	3	*CFTR*
7	q35	144,922,849	150,951,819	89	2	
8	q12.1	58,780,480	65,128,132	78	2	
9	p24.2	3,939,996	12,907,793	98	2	
10	q23.31	116,005,494	124,214,355	120	2	*EMX2*
12	p13.32	3,780,336	7,918,460	89	2	
12	q13.13	58,000,215	68,228,170	56	2	
12	q13.3	103,118,607	113,263,934	45	3	
13	q12.13	33,381,720	34,694,189	32	4	
13	q22.3	77,503,539	87,943,460	23	2	
14	q31.3	92,919,833	94,993,744	45	2	
15	q22.2	60,644,347	68,204,581	67	2	
17	q11.1	35,694,046	41,797,254	34	2	
18	p11.22	8,993,423	12,697,711	60	3	
18	q22.1	66,236,242	74,326,105	78	2	
20	q11.21	45,391,728	46,347,251	24	2	
20	q13.12	50,008,791	53,427,207	12	2	
22	q11.21	44,669,027	45,906,107	10	2	
X	q11.1	61,932,503	66,974,524	45	8	
X	q13.1	71,819,690	77,853,204	32	2

## Discussion

The pathogenesis of MRKH in humans is largely unknown, but could include genetic (germline or somatic cell mutations), epigenetic, and/or environmental etiologies. There is evidence supporting a genetic etiology, as demonstrated by families with more than one affected proband [7]. Although twin studies in which monozygotic twins show greater concordance vs. dizygotic twins support a genetic component [26], there have been few studies in MRKH. Those small number of monozyogotic twins have been discordant for MRKH [27–29]. The genetic basis of MRKH is largely unknown except for occasional heterozygous *WNT4* or *HNF1B* mutations [8, 12]. Many investigators have performed CMA on MRKH patients and have suggested possible pathogenic CNVs [19, 30]. It is interesting to note that these CNVs may be found in isolated MRKH (type I) or those with associated anomalies (type II) [19, 30]. In the present study, we found five CNVs in four patients with type I MRKH, three of whom were products of consanguineous parents. This is consistent with the overall 75% rate of consanguinity in our study. The 21% prevalence of CNVs in our largely consanguineous Turkish population does not seem to differ with the prevalence in studies of Europe and North America, which range from 16 to 46% (26% overall in four studies) [17, 19–21].

The previously reported 16p11.2 deletion was observed in one patient. Patients with microdeletions at 16p11.2 may show variable clinical features including autism [31], epilepsy, global developmental delay, dysmorphism, behavioral problems, abnormal head size [32], and obesity [32]. Microdeletions at 16p11.2 are also common in patients with type I and type II MRKH [19, 21]. This region contains more than 30 genes. The T Box 6 (*TBX6*) gene located in this region represents an attractive candidate gene, but to date, no causative mutations have been confirmed. This same patient had an Xq25 deletion, which contains one gene—*ACTRT1* (actin-related protein T1), which has no proven relation to MRKH at this time. Two other type I patients had CNVs containing only one gene—a 16p13.3 deletion (*RBFOX1*) and a 13q14.11 duplication (*FOXO1*). The remaining type I patient had a 1p31.1 duplication containing three genes (*ST6GALNAC3, MSH4,* and *ASB17*). The 16p13.3 region and the *RBFOX1* gene have been implicated in autism; *FOXO1* is a transcription factor; and *ST6GALNAC3* is expressed in the reproductive tract. *MSH4* is a member of the DNA mismatch repair mutS family necessary for reciprocal recombination and proper segregation of homologous chromosomes at meiosis I. *ASB17,* which is highly expressed in the testis, is a component of E3 ubiquitin-protein ligase complex that mediates the ubiquitination and subsequent proteasomal degradation of target proteins.

The significance of these CNVs is uncertain at this time, but it is unlikely that the 16p13.3 deletion is involved in the pathogenesis of MRKH because it occurs frequently in both the DGV and Decipher databases. Alternatively, the 16p11.2 CNV has been previously reported in MRKH, and large CNVs similar in size are infrequent in these two databases. The other three are potentially pathogenic CNVs—Xq25, 13q14.11, and 1p31.1.

When the literature is examined, chromosomal regions 17q12, 16p11, 22q11, and 1q21.1 harbor some of the more common CNVs in MRKH [16–21]. Deletions of 17q12 generally range from 1.2–1.8 Mb in size and contain ~ 17–20 genes. Known causative gene and transcription factor *HNF1B* resides within this region and heterozygous mutations result in maturity onset diabetes of the young type 5 (MODY5). Associated findings with this phenotype may include renal cysts and Müllerian aplasia [12]. *LHX1* is another potential causative gene within this region, as the knockout mouse has a phenotype consistent with MRKH. However, there are currently no clear causative human *LHX1* mutations, confirmed by in vitro analyses supported by family studies [2, 33]. We have recently performed Sanger DNA sequencing on 100 North American and Turkish MRKH women and none had small insertion/deletions or point mutations in *WNT4*, *LHX1*, or *HNF1B* suggesting variants are rare in these genes [2]. The 22q11 region is involved in the DiGeorge phenotype and other associated disorders, while deletions or duplications of 1q21.1 have been identified in ttype I MRKH. However, their significance to the pathophysiology of MRKH is unknown at this time [30].

Copy number variants are typically heterozygous [2], but since consanguineous marriages are common in Turkey, we sought to determine if MRKH patients had large regions of homozygosity (ROH). Turkish patients in the current study consisted of Anatolian-origin Caucasians, who are predominantly from Antalya, Turkey. As reported by Alper et al. in 2004, the rate of consanguineous marriages in the province of Antalya was found to be 33.9% [34]. People in this region have a greater risk of autosomal recessively inherited genetic diseases. Analysis of ROH may provide a good starting point to determine the genetic basis of disease in the offspring of such consanguineous families. Ours is the first study, to our knowledge, to examine ROH analysis in consanguineous MRKH families by CMA.

It is interesting that nearly three quarters of our Turkish MRKH patients demonstrated consanguinity, as defined by having parents that were third degree relatives or closer. In all eight of our patients who stated their parents were first cousins, all were second or first degree relatives. For the remaining 11 MRKH patients who did not know whether consanguinity was present, 7/11 had parents that were third or second degree relatives. Therefore, the chance of consanguinity was greater in MRKH patients than reported for Anatolian people in general, which suggests that autosomal recessive loci could be responsible for some causes of MRKH.

Further supporting consanguinity, there were 42 regions across the genome in which at least two MRKH patients had overlapping homozygous genomic regions, most frequently chromosomes 2, 3, and 4. None of the shared regions included the 17q12 or 16p11.2 CNVs, but did include 22q11.21. When putative candidate genes from the literature are surveyed, either based upon probable function and/or animal models, eight genes (*RBM8A, CMTM7, CCR4, TRIM71, CNOT10, TP63, EMX2,* and *CFTR*) reside within these shared regions, which could suggest a role in MRKH and a possible founder effect if mutations are discovered (Table 5).

**Table 5:** Genes implicated in mullerian development are shown from mouse and human studies, including the 3;16 translocation. Genes in bold reside within regions of homozygosity in ≥ 2 MRKH patients

Mouse studies	*Wnt4, Lhx1,* ***Emx2***, *Pbx2 Wnt9b*, *Pax2*, *Wnt5a*, *Rar, Rxr*, ***Tp63***, *Wnt7a*, *Hoxa9, Hoxa10, Hoxa11, Hoxa12, Hoxa13*
Human Studies	*WNT4, HNF1B, ZNHIT3, WT1,* ***CFTR*** *, WNT7A, GALT, HOXA7, PBX1, HOXA10, AMH, AMHR, RARG, RXRA, CTNNB1, PAX2, LAMC1, DLGH1, SHOX,MMP14, LRP10, WNT9B PBX1, LHX1,* ***RBM8A*** *, TBX6*
Human Translocation	***CMTM7, CCR4*** *, IL32, MEFV,* ***TRIM71, CNOT10*** *, ZNF200, OR1F1, ZNF213, ZNF205*

The inheritance of MRKH is most likely to be autosomal dominant for most of the world based upon heterozygous single gene mutations and heterozygous CNVs. However, the large percentage of consanguinity and shared regions of homozygosity in Turkish MRKH patients suggest the existence of an autosomal recessive form. Ideally, homozygosity mapping followed by whole exome sequencing to pinpoint the causative genes should be done in more patients and their family members to narrow down candidate genomic regions for MRKH. However, our results provide additional candidate genes to study, and we suggest that there may be autosomal recessive causes of MRKH that could be identified in consanguineous Turkish families.

## Conclusion

CNVs were identified in approximately 20% of Turkish MRKH patients, but it is unknown if they are causative. It is interesting that the 16p11.2 deletion CNV seen in other populations was also found in a Turkish MRKH patient. Our findings suggest that Turkish MRKH patients have a greater chance of consanguinity than the general Anatolian Turkish population. In contrast to other reports suggesting autosomal dominant inheritance of MRKH, the extremely high rate of shared regions of homozygosity suggests that inheritance of some cases of MRKH in Turkey could be autosomal recessive.

## References

[1] Acien P, Acien M, Sanchez-Ferrer M (2004). Complex malformations of the female genital tract. New types and revision of classification. Hum Reprod.

[2] Williams LS, Demir Eksi D, Shen Y, Lossie AC, Chorich LP, Sullivan ME (2017). Genetic analysis of Mayer-Rokitansky-Kuster-Hauser syndrome in a large cohort of families. Fertil Steril.

[3] Oppelt PG, Lermann J, Strick R, Dittrich R, Strissel P, Rettig I (2012). Malformations in a cohort of 284 women with Mayer-Rokitansky-Kuster-Hauser syndrome (MRKH). Reprod Biol Endocrinol.

[4] Bean EJ, Mazur T, Robinson AD (2009). Mayer-Rokitansky-Kuster-Hauser syndrome: sexuality, psychological effects, and quality of life. J Pediatr Adolesc Gynecol.

[5] Reindollar RH, Byrd JR, McDonough PG (1981). Delayed sexual development:study of 252 patients. Am J Obstet Gynecol.

[6] Simpson JL (2014). Genetics of female infertility due to anomalies of the ovary and mullerian ducts. Methods Mol Biol.

[7] Herlin M, Hojland AT, Petersen MB (2014). Familial occurrence of Mayer-Rokitansky-Kuster-Hauser syndrome: a case report and review of the literature. Am J Med Genet A.

[8] Biason-Lauber A, De Filippo G, Konrad D, Scarano G, Nazzaro A, Schoenle EJ (2007). WNT4 deficiency--a clinical phenotype distinct from the classic Mayer-Rokitansky-Kuster-Hauser syndrome: a case report. Hum Reprod.

[9] Biason-Lauber A, Konrad D, Navratil F, Schoenle EJ (2004). A WNT4 mutation associated with Mullerian-duct regression and virilization in a 46,XX woman. N Engl J Med.

[10] Philibert P, Biason-Lauber A, Gueorguieva I, Stuckens C, Pienkowski C, Lebon-Labich B (2011). Molecular analysis of WNT4 gene in four adolescent girls with mullerian duct abnormality and hyperandrogenism (atypical Mayer-Rokitansky-Kuster-Hauser syndrome). Fertil Steril.

[11] Philibert P, Biason-Lauber A, Rouzier R, Pienkowski C, Paris F, Konrad D (2008). Identification and functional analysis of a new WNT4 gene mutation among 28 adolescent girls with primary amenorrhea and mullerian duct abnormalities: a French collaborative study. J Clin Endocrinol Metab.

[12] Lindner TH, Njolstad PR, Horikawa Y, Bostad L, Bell GI, Sovik O (1999). A novel syndrome of diabetes mellitus, renal dysfunction and genital malformation associated with a partial deletion of the pseudo-POU domain of hepatocyte nuclear factor-1beta. Hum Mol Genet.

[13] Kucheria K, Taneja N, Kinra G (1988). Autosomal translocation of chromosomes 12q & 14q in mullerian duct failure. Indian J Med Res.

[14] Amesse L, Yen FF, Weisskopf B, Hertweck SP (1999). Vaginal uterine agenesis associated with amastia in a phenotypic female with a de novo 46,XX,t(8;13)(q22.1;q32.1) translocation. Clin Genet.

[15] Williams LS, Kim HG, Kalscheuer VM, Tuck JM, Chorich LP, Sullivan ME (2016). A balanced chromosomal translocation involving chromosomes 3 and 16 in a patient with Mayer-Rokitansky-Kuster-Hauser syndrome reveals new candidate genes at 3p22.3 and 16p13.3. Mol Cytogenet.

[16] Cheroki C, Krepischi-Santos AC, Rosenberg C, Jehee FS, Mingroni-Netto RC, Pavanello Filho I (2006). Report of a del22q11 in a patient with Mayer-Rokitansky-Kuster-Hauser (MRKH) anomaly and exclusion of WNT-4, RAR-gamma, and RXR-alpha as major genes determining MRKH anomaly in a study of 25 affected women. Am J Med Genet A.

[17] Cheroki C, Krepischi-Santos AC, Szuhai K, Brenner V, Kim CA, Otto PA (2008). genomic imbalances associated with mullerian aplasia. J Med Genet.

[18] Morcel K, Watrin T, Pasquier L, Rochard L, Le Caignec C, Dubourg C (2011). Utero-vaginal aplasia (Mayer-Rokitansky-Kuster-Hauser syndrome) associated with deletions in known DiGeorge or DiGeorge-like loci. Orphanet J Rare Dis.

[19] Nik-Zainal S, Strick R, Storer M, Huang N, Rad R, Willatt L (2011). High incidence of recurrent copy number variants in patients with isolated and syndromic Mullerian aplasia. J Med Genet.

[20] Ledig S, Schippert C, Strick R, Beckmann MW, Oppelt PG, Wieacker P (2011). Recurrent aberrations identified by array-CGH in patients with Mayer-Rokitansky-Kuster-Hauser syndrome. Fertil Steril.

[21] Sandbacka M, Laivuori H, Freitas E, Halttunen M, Jokimaa V, Morin-Papunen L (2013). TBX6, LHX1 and copy number variations in the complex genetics of Mullerian aplasia. Orphanet J Rare Dis.

[22] McGowan R, Tydeman G, Shapiro D, Craig T, Morrison N, Logan S (2015). DNA copy number variations are important in the complex genetic architecture of mullerian disorders. Fertil Steril.

[23] Chen MJ, Wei SY, Yang WS, Wu TT, Li HY, Ho HN (2015). Concurrent exome-targeted next-generation sequencing and single nucleotide polymorphism array to identify the causative genetic aberrations of isolated Mayer-Rokitansky-Kuster-Hauser syndrome. Hum Reprod.

[24] Lahiri DK, Nurnberger JI (1991). A rapid non-enzymatic method for the preparation of HMW DNA from blood for RFLP studies. Nucleic Acids Res.

[25] Sund KL, Zimmerman SL, Thomas C, Mitchell AL, Prada CE, Grote L (2013). regions of homozygosity identified by SNP microarray analysis aid in the diagnosis of autosomal recessive disease and incidentally detect parental blood relationships. Genetics in medicine : official journal of the American College of Medical Genetics.

[26] Kondo S, Schutte BC, Richardson RJ, Bjork BC, knight AS, Watanabe Y (2002). mutations in IRF6 cause van der Woude and popliteal pterygium syndromes. Nat Genet.

[27] Duru UA, Laufer MR (2009). Discordance in Mayer-von Rokitansky-Kuster-Hauser syndrome noted in monozygotic twins. J Pediatr Adolesc Gynecol.

[28] Milsom SR, Ogilvie CM, Jefferies C, Cree L (2015). discordant Mayer-Rokitansky-Kuster-Hauser (MRKH) syndrome in identical twins - a case report and implications for reproduction in MRKH women. Gynecological endocrinology : the official journal of the International Society of Gynecological Endocrinology.

[29] Rall K, Eisenbeis S, Barresi G, Ruckner D, Walter M, Poths S (2015). Mayer-Rokitansky-Kuster-Hauser syndrome discordance in monozygotic twins: matrix metalloproteinase 14, low-density lipoprotein receptor-related protein 10, extracellular matrix, and neoangiogenesis genes identified as candidate genes in a tissue-specific mosaicism. Fertil Steril.

[30] Layman LC (2014). The genetics of mullerian aplasia. Expert Rev Endocrinol Metab.

[31] Weiss LA, Shen Y, Korn JM, Arking DE, miller DT, Fossdal R *et al.* association between microdeletion and microduplication at 16p11.2 and autism. N Engl J Med 2008;358:667–675. 10.1056/NEJMoa075974 18184952

[32] Walters RG, Jacquemont S, Valsesia A, de Smith AJ, martinet D, Andersson J (2010). a new highly penetrant form of obesity due to deletions on chromosome 16p11.2. Nature.

[33] Zhang W, Zhou X, Liu L, Zhu Y, Liu C, Pan H (2017). Identification and functional analysis of a novel LHX1 mutation associated with congenital absence of the uterus and vagina. Oncotarget.

[34] Alper OM, Erengin H, Manguoglu AE, Bilgen T, Cetin Z, Dedeoglu N (2004). Consanguineous marriages in the province of Antalya, Turkey. Ann Genet.

